# Four raised to one equals one: A genetic approach to the *Pseudolaelia vellozicola* complex does not follow a math rule

**DOI:** 10.1002/ece3.6148

**Published:** 2020-05-05

**Authors:** Alison Gonçalves Nazareno, Luiz Menini Neto, Renata Santiago de Oliveira Buzatti, Cássio van den Berg, Rafaela Campostrini Forzza

**Affiliations:** ^1^ Department of Genetics, Ecology and Evolution Federal University of Minas Gerais Belo Horizonte Brazil; ^2^ Rio de Janeiro Botanical Garden Rio de Janeiro Brazil; ^3^ Department of Biological Sciences Universidade Estadual de Feira de Santana Feira de Santana Brazil

**Keywords:** genetic structure, intersimple sequence repeat, Orchidaceae, *Pseudolaelia aguadocensis*, *Pseudolaelia oliveirana*, *Pseudolaelia regentii*, *Pseudolaelia vellozicola*

## Abstract

*Pseudolaelia* is a genus endemic to the eastern Brazilian Atlantic Forest, consisting of 12 accepted species. Some *Pseudolaelia* species, such as *P. vellozicola*, *P. aguadocensis*, *P*. *oliveirana*, and *P*. *regentii*, referred to here as the PV complex, present extensive intra‐ and interpopulation morphological polymorphism, raising uncertainty regarding their circumscriptions. Although previous morphological analyses were used to solve the generic boundaries in the PV complex, persuasive genetic evidence is lacking. In order to test the hypothesis that the group under investigation contains only one taxon, amplification profiles of five intersimple sequence repeat (ISSR) markers were used to evaluate genetic diversity, genetic structure, and the relationships among the PV complex species. A total of 134 reproductive individuals were sampled in eight insular populations. Intrapopulation genetic analysis indicated low levels of genetic diversity. Analysis of genetic structure revealed that each of the eight sample locations can be considered unique biological populations as they are highly differentiated from each other. The Mantel test showed a high and positive correlation between genetic and geographic distance (*r* = .841, *p* < .002), indicating isolation by distance. The results are consistent with that expected for plants with insular geographical distribution. When testing for the null hypothesis, the low levels of genetic variation among species (*F*_CT _= 0.155) suggest that the populations constitute only one highly polymorphic species with a wide distribution.

## Graphical abstract

Analysis of genetic structure revealed that each of the eight sample localities can be considered unique biological populations as they are highly differentiated from each other. The results are consistent with that expected for plants with insular geographical distribution. When testing for the null hypothesis, the low levels of genetic variation among species suggest that the populations constitute only one highly polymorphic species with a wide distribution.

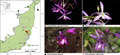

## 1 INTRODUCTION

The orchid family (Orchidaceae) is one of the largest families of flowering plants, with roughly 26,000 species distributed worldwide, the majority of which occur in tropical regions. In the Neotropics, Brazil is one of the richest countries in Orchidaceae, with over 2,420 species distributed across 207 genera (Barros et al., 2015). *Pseudolaelia* Porto & Brade (Orchidaceae, Laeliinae) is a small genus endemic to eastern Brazil, consisting of 12 accepted species (Menini Neto, Forzza, & Berg, 2013). Orchids of the genus *Pseudolaelia* occur predominantly as an epiphyte on *Vellozia* (a fibrous shrubby plant species of the Velloziaceae family), growing in granitic and gneissic outcrops (inselbergs) of the Brazilian Atlantic Forest biome and quartzite outcrops of the Cerrado and Caatinga grasslands (Menini Neto, 2011). *Pseudolaelia* is a monophyletic genus that is part of a basal clade of the subtribe Laeliinae called Isabelia Alliance (van den Berg et al., 2009). Besides small population sizes and habitat specificity, all *Pseudolaelia* species are threatened to some degree due to habitat degradation caused by mining and uncontrolled tourism (Menini Neto, 2011).

The orchid genus *Pseudolaelia* is characterized by homoblastic, fusiform, or piriform pseudobulbs separated by a long rhizome, with cataphylls that disintegrate into fibers. Its indeterminate inflorescence is long, simple, or compound, with pink, yellow, white, or cream maculate or concolorous flowers. Flowers present a simple labellum or are often trilobate, sometimes entire, with entire or erose margins, and the cuniculus present (Menini Neto, 2011). Although the majority of species morphologies for *Pseudolaelia* are quite homogeneous, a taxonomic revision based on analyses of herbarium specimens and field studies for the genus revealed remarkable floral polymorphism for *Pseudolaelia vellozicola* (Hoehne) Porto & Brade populations (Menini Neto et al., 2013). These results, along with recent descriptions of some very similar taxa, viz. *Pseudolaelia aguadocensis* Campacci (Campacci, 2016), *Pseudolaelia regentii* V.P.Castro & Marçal (Castro Neto & Marçal, 2007), and *Pseudolaelia oliveirana* V.P.Castro & Chiron (Castro Neto & Chiron, 2009), have made the taxonomical definition of *P. vellozicola* species more complex. However, Menini Neto, Berg, and Forzza (2019) carried out a morphological study to better understand if the morphological variability found in *P. vellozicola* is due to its insular distribution (which would synonymize *P. vellozicola*, *P. aguadocensis*, *P. oliveirana*, and *P. regentii*), or whether they are different species constituting a complex. Despite the large interpopulation floral variation (that may originate from the relative geographic isolation among insular populations), Menini Neto et al. (2019) reported that there are no strong morphological discontinuities among the populations of *P. vellozicola*, *P. aguadocensis*, *P. oliveirana,* and *P. regentii*. The species hypothesis resulting from this previous study (Menini Neto et al., 2019) indicated that the morphological differences are insufficient to separate these taxa, suggesting that the PV complex consists of only one species.

Although morphological studies fulfill an important role in systematic identification (Duminil & Di Michele, 2009; Henderson, 2006; Rohlf, 1990), the use of molecular tools has contributed significantly to the identification of plants and animals (Duminil & Di Michele, 2009; Gaiero, Mazzella, Agostini, Bertolazzi, & Rossato, 2011; Kiani, Memariani, & Zarghami, 2012; Rodrigues et al., 2015). Intersimple sequence repeat (ISSR) markers are an extremely useful genetic tool that can be used in taxonomic comparisons and to determine intra‐ and interpopulation levels of genetic variability. Furthermore, ISSR patterns are species‐specific and, as such, enable the identification of profiles in related plant species (Bozchaloyi, Sheidai, Keshavarzi, & Noormohammadi, 2018; Fajardo, Vieira, & Molina, 2014; Zietkiewicz, Rafalski, & Labuda, 1994). In species with unclear taxonomical definitions, as with some *Pseudolaelia* species, the use of ISSR markers can help to solve this puzzle. Given the taxonomic challenge of distinguishing *P. aguadocensis*, *P*. *oliveirana*, *P*. *regentii,* and *P. vellozicola,* this study sought to test the hypothesis that the group under investigation contains only one taxon. For this, amplification profiles of five intersimple sequence repeat (ISSR) markers were used to evaluate genetic diversity, genetic structure, and the relationships among the studied populations and species of the PV complex.

## 2 MATERIALS AND METHODS

### 2.1 Study area, population sampling, and DNA extraction

*Pseudolaelia* occurs in a particular class of residual landforms called inselbergs, which are isolated rock outcrops (height > 100 m) that rise abruptly above the surrounding grasslands (Lima & Corrêa‐Gomes, 2015). Inselbergs may be isolated or grouped, separated by only a few or many kilometers across the landscape. Ecologically, they are characterized as having harsh environments, including a high degree of insolation, high rates of evaporation, local and very restricted soil occurrence, and water scarcity, all of which favor the development and occurrence of a large number of endemic species (Porembski, 2007).

**Figure 1:**
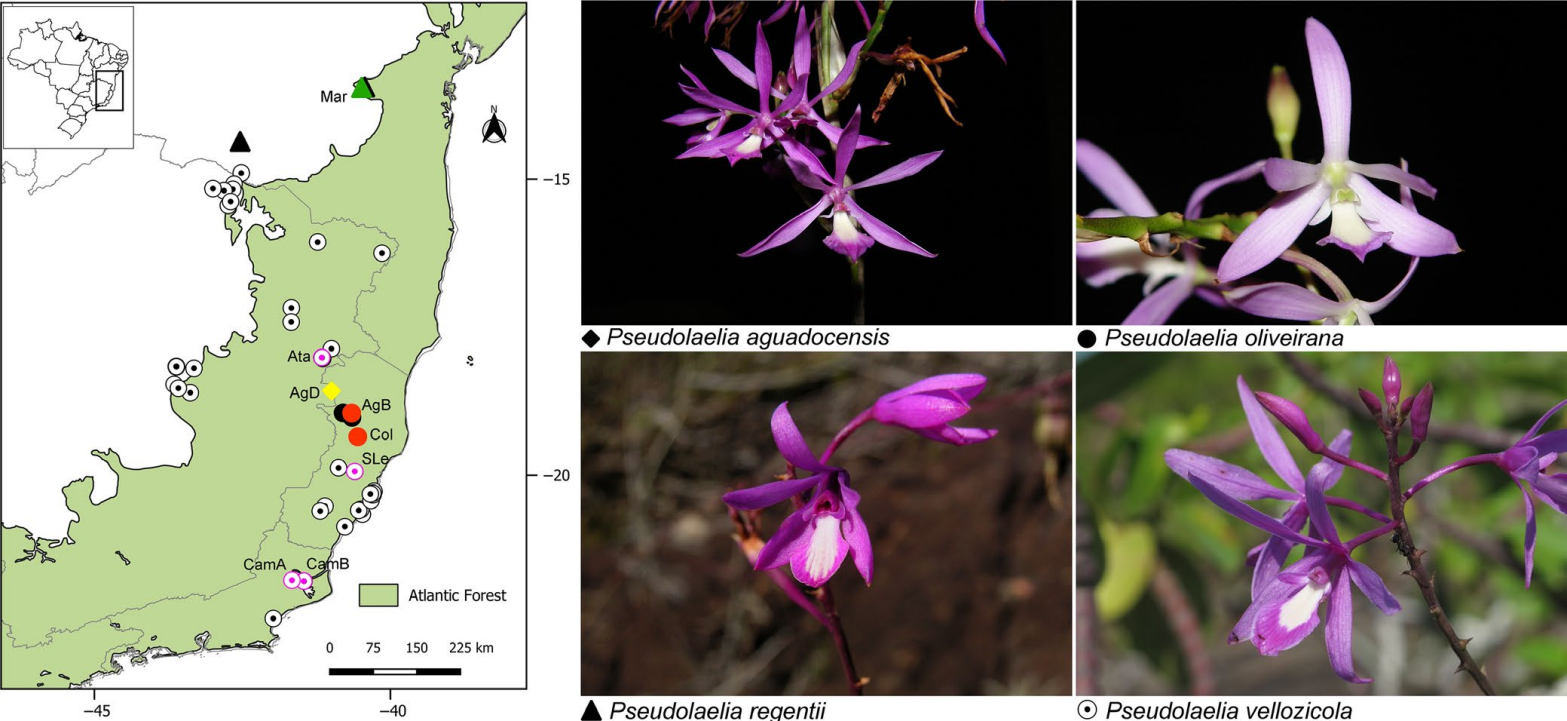
Sampling localities, geographic distribution, and pictures of the putative species (*Pseudolaelia aguadocensis*, *P*. *oliveirana*, *P*. *regentii*, and *P. vellozicola*) that comprise the PV complex are showed. Colors used for coding the eight sampled localities: yellow to *P. aguadocensis*, red to *P. oliveirana*, green to *P. regentii*, and pink to *P. vellozicola*

Due to its endemism and rarity, eight locations were sampled across the entire geographic distribution of the PV complex (Figure 1). In order to avoid taxonomic identification errors, the sampling included the same localities that were previously used in a taxonomic revision of the genus (Menini Neto et al., 2013). A total of 134 reproductive individuals of the PV complex were sampled, and the sample size at each locality varied according to the characteristics of the population (Table 1). Specifically, the sampling included one locality (AgD) related to *P. aguadocensis*, two localities (AgB and Col) related to *P. oliveirana*, one locality (Mar) related to *P. regentii,* and four localities (Ata, CamA, CamB, and SLe) related to *P. vezicolla* (Figure 1). Voucher specimens from the sample localities were deposited in one of the following herbaria: Federal University of Juiz de Fora (CESJ); State University of Feira de Santana (HUEFS); Mello Leitão Biological Museum (MBML); or Botanical Garden of Rio de Janeiro (RB; Table 1).

**Table 1:** Locality, abbreviation, coordinates, elevation, sample size, and voucher information for each studied locality of the *Pseudolaelia vellozicola* complex

Locality–State (abbreviation)	Latitude (S)–Longitude (W)	Altitude (m)	Sample size	Voucher
Água Doce do Norte–ES (AgD)	18°34′28″S–40°59′36″W	610	17	Fontana 5053 (MBML)
Águia Branca–ES (AgB)	18°58′59″S–40°39′59″W	360	16	Fontana 5040 (MBML)
Ataléia–MG (Ata)	18°01′48″S–41°08′32″W	330	20	Menini Neto 754 (CESJ, RB)
Campos dos Goitacazes–RJ (CamA)	21°47′19″S–41°27′27″W	50	20	Menini Neto 520 (CESJ, RB)
Campos dos Goitacazes–RJ (CamB)	21°48′00″S–41°28′08″W	30	11	—
Colatina–ES (Col)	19°20′53″S–40°33′03″W	524	20	Fontana 5021(MBML)
Maracás–BA (Mar)	13°27′00″S–40°28′59″W	900	15	Menini Neto 769 (CESJ, RB, HUEFS)
Santa Leopoldina–ES (SLe)	20°08′58″S–40°37′29″W	478	15	Fontana 4904 (MBML)

Note

Voucher location: Federal University of Juiz de Fora (CESJ); State University of Feira de Santana (HUEFS); Mello Leitão Biological Museum (MBML); or Botanical Garden of Rio de Janeiro (RB).

Abbreviations: BA, Bahia; ES, Espírito Santo; MG, Minas Gerais; RJ, Rio de Janeiro.

Leaf fragments from each individual were collected and stored in 2.5‐ml plastic tubes containing a gel prepared with 2 g of cetyltrimethylammonium bromide (CTAB), 35 g of NaCl, and distilled water to a final volume of 100 ml. Samples were kept refrigerated at 4°C until DNA extraction. Genomic DNA was isolated using the CTAB protocol (Doyle & Doyle, 1987).

### 2.2 Primer screening and PCR amplification

We tested a set of 20 ISSR primers, previously described by Wolfe (2000). The ISSR primer pairs used in this study were UBC814, UBC843, UBC844, UBC898, UBC899, UBC901, UBC902, AW3, BECKY, CHRIS, DAT, GOOFY, M1, M2, R7, JOHN, MANNY, MAO, OMAR, and TERRY. PCR amplifications were performed in a Thermocycler GeneAmp PCR System 9700 (Applied Biosystems) using 18‐µl reactions with 20 ng of genomic DNA, 2.0 µl 10× PCR buffer (Phoneutria), 1.7 mM dNTPs, 2.5 mM of MgCl_2_, 1 U of Taq polymerase (Phoneutria), 0.36 µM primer, and double‐distilled water. The program consisted of an initial denaturation of 94°C for 110 s, followed by 35 cycles of 40 s at 94°C, 45 s at 45°C (annealing temperature), 72°C extension for 110 s, and a final extension of 72°C for 7 min. Amplifications with each ISSR primer were repeated to ensure reproducibility. Amplicons were separated on 1.5% agarose gels stained with ethidium bromide and verified with UV light. The molecular size of the fragments was identified with the aid of a 100‐bp DNA ladder. Genotyping was performed manually by comparing banding patterns on the gels.

### 2.3 Data analyses

After electrophoresis, clear and intense bands were scored and transformed into binary character matrices, indicating the presence (1) or absence (0) of amplified fragments; amplicons of similar molecular size with the same primer were assumed to be homologous. To evaluate genetic diversity, the presence/absence matrix was analyzed using the POPGENE v. 1.32 software (Yeh, Yang, Boyle, Ye, & Mao, 1997) and the following parameters were estimated: percentage of polymorphic loci (*P*); allele frequencies; Nei's genetic diversity (*h*; Nei, 1973); and Shannon diversity index (*I*). To verify the distribution of private loci across the PV complex, we constructed a Venn diagram using R (R Core Team, 2018). In addition, we estimated genetic diversity indices considering the hypothesis of differentiation between the taxa that comprise the PV complex. For *H*_E_ and *I*, the 95% confidence intervals were calculated to help evaluate differences between estimated means for all species.

In order to infer population structure, a model‐based approach was performed using the STRUCTURE 2.2 program (Pritchard, Stephens, & Donnelly, 2000). This method accounts more accurately for the inherent ambiguity of recessive (absent) alleles in ISSR marker data sets. Ten independent runs were performed for *K* varying from one to nine. Each run consisted of 500,000 Markov chain Monte Carlo (MCMC) iterations, with an initial burn‐in of 100,000 iterations. The analysis was performed assuming that the allele frequencies in different populations can be correlated with one another and that alleles carried at a particular locus by a particular individual originated in some unknown population (admixture model). To infer the most probable *K* to explain the data, we calculated the average of each *K* likelihood value, log of probability (LnP(D)), through all runs, as suggested by Pritchard et al. (2000), and the Δ*K* statistic was calculated according to Evanno, Regnaut, and Goudet (2005).

Population genetic structure and differentiation were assessed through Analysis of Molecular Variance (AMOVA; Excoffier & Lischer, 2010) using the program ARLEQUIN version 3.5.1.2. First, the partition of genetic variance was evaluated within and among sampled localities to assess the effect of geographical distance. Secondly, we grouped the sample localities based on current species definitions.

We also estimated the pairwise *F*_ST_ among populations using ARLEQUIN version 3.5.1.2 (Excoffier & Lischer, 2010) with significance obtained through 10,000 randomizations. In order to investigate the correlation between genetic and geographic distance, we used the Mantel test to determine if the pattern met the expectation of decreased genetic similarity with increased geographic distance, or isolation by distance (IBD). Using 10,000 permutation tests of significance for the correlation coefficient, a single Mantel test between a matrix of pairwise genetic distance [*F*_ST_/(1–*F*_ST_)] and a matrix of Euclidian distance was performed using ARLEQUIN version 3.5.1.2 (Excoffier & Lischer, 2010).

## 3 RESULTS

Five of the 20 tested primers showed high polymorphism and resulted in 94 fragments varying from 100 to 2,000 bp. Population‐level genetic parameters are summarized in Table 2. Each primer amplified between 14 and 22 fragments, and the ISSR population analyses revealed low levels of within‐population genetic diversity, with the percentage of polymorphic loci (*P*) ranging from 11.8% to 43.4% (Table 2). The values for Nei's genetic diversity (*H*_E_) and Shannon index (*I*) were low (Table 2), ranging from *H*_E_ = 0.048 and *I* = 0.069 (Mar) to *H*_E_ = 0.137 and *I* = 0.203 (Ata).

**Table 2:** Estimates of genetic diversity for eight populations of the *Pseudolaelia vellozicola* complex based on five intersimple sequence repeat (ISSR) markers

Locality	*N*	*P* (%)	*H*_E_ (±*SD*)	*I* (±*SD*)
AgB	17	22.4	0.075 (±0.154)	0.113 (±0.226)
AgD	23	30.3	0.093 (±0.164)	0.142 (±0.239)
Ata	29	38.2	0.137 (±0.197)	0.203 (±0.282)
CamA	15	19.7	0.062 (±0.140)	0.096 (±0.207)
CamB	17	22.4	0.074 (±0.151)	0.113 (±0.226)
Col	33	43.4	0.129 (±0.177)	0.200 (±0.258)
Mar	9	11.8	0.048 (±0.140)	0.069 (±0.198)
SLe	23	30.3	0.099 (±0.170)	0.150 (±0.247)
Mean	20.75	27.3	0.106 (±0.054)	0.163 (±0.088)
*Pseudolaelia aguadocensis*	23	30.3	0.093 (±0.164)	0.142 (±0.239)
*Pseudolaelia oliveirana*	41	53.6	0.159 (±0.180)	0.247 (±0.263)
*Pseudolaelia regentii*	9	11.8	0.048 (±0.139)	0.069 (±0.198)
*Pseudolaelia vellozicola*	62	81.6	0.230 (±0.176)	0.357 (±0.246)

Note

*N*, number of polymorphic loci; *P*, percentage of polymorphic loci, *H*_E_, expected heterozygosity; *I*, Shannon diversity index. Standard deviation in parentheses. For locality name see Table 1.

**Figure 2:**
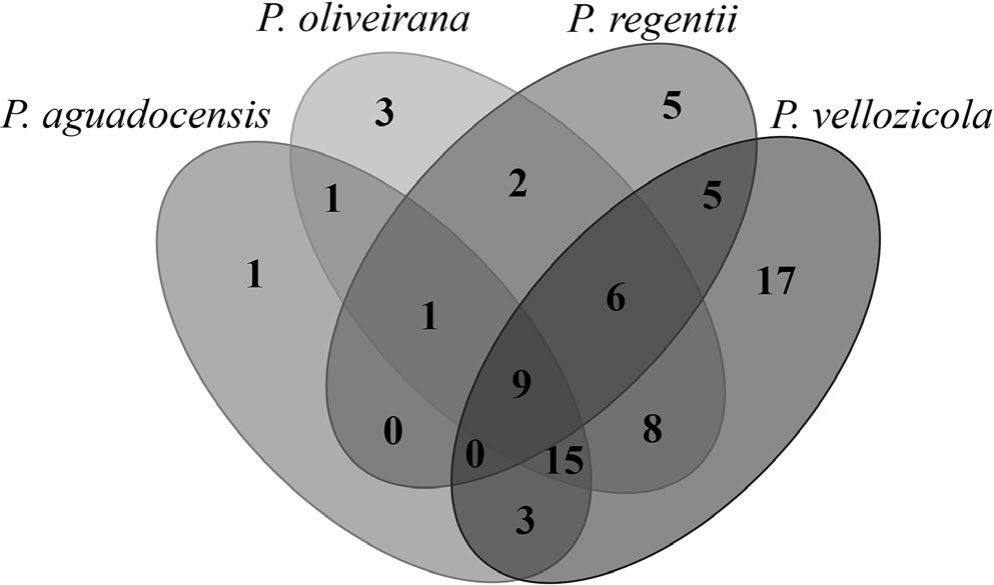
Venn diagram representing the distribution of private loci across the putative species (*Pseudolaelia aguadocensis*, *P*. *oliveirana, P*. *regentii,* and *P. vellozicola*) that comprise the PV complex

At the species level, the percentage of polymorphic loci was 30.3 for *P. aguadocensis*, 53.6% for *P. oliveirana*, 11.8% for *P. regentii*, and 81.6% for *P. vellozicola* (Table 2). The mean Nei's genetic diversity varied from 0.048 (*P. regentii*) to 0.230 (*P. vellozicola*), and the mean values for Shannon index ranged from 0.069 (*P. regentii*) to 0.357 (*P. vellozicola*; Table 2). At the species level, we found no significant differences for the studied genetic diversity indexes: *P. aguadocensis* (*H*_E_—95% CI 0.034, 0.152; *I*—95% CI 0.056, 0.228); *P. oliveirana* (*H*_E_—95% CI 0.106, 0.212; *I*—95% CI 0.169, 0.325); *P. regentii* (*H*_E_—95% CI −0.004, 0.100; *I*—95% CI −0.005, 0.143); *P. vellozicola* (*H*_E_—95% CI 0.141, 0.319; *I*—95% CI 0.223, 0.481). In terms of the number of private loci across the PV complex, we verified some differences among *Pseudolaelia* species (see Venn diagram, Figure 2), with *P. vellozicola* showing the greatest number of private loci (*n* = 17) compared to *P. aguadocensis* (*n* = 1), *P. oliveirana* (*n* = 3), and *P. regentii* (*n* = 5; Figure 2).

**Figure 3:**
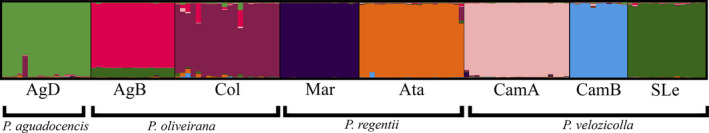
Bar plot representation of the eight genetic populations of the *Pseudolaelia vellozicola* complex inferred using Bayesian analysis (STRUCTURE) of 134 individuals from eight localities

Using Bayesian inference in the STRUCTURE program, the analysis of genetic structure produced an optimal value of *K* = 8. This result indicates that each sampling locality can be considered a unique population (Figure 3, Figure S1a,b). The significant variation in genetic structure across sampling localities was also evident in the hierarchical multilocus evaluation of genetic differentiation performed using AMOVA, which indicates that a greater proportion of the overall genetic variation exists among populations (Table 3). At the species level, only 15.5% of the overall genetic variation was observed among *Pseudolaelia* species (*F*_CT_ = 0.155; Table 3), with the variation among localities within species explaining the greatest proportion of overall genetic variation (52%; Table 3).

**Table 3:** Results of the Analysis of Molecular Variance (AMOVA) for the *Pseudolaelia vellozicola* complex

Source of variation	Variance component	Percentage of variance	Fixation index (*P*)
Genetic variation			
Among localities	7.34	66.29	***F*_ST_ = 0.663**
Within localities	3.73	33.71	
Genetic variation considering four species			
Among species	1.79	15.52	*F*_CT_ = 0.155
Among localities within species	5.99	52.05	***F*_SC_ = 0.616**
Within localities	3.73	32.43	***F*_ST_ = 0.676**

Note

Significant values for fixation indexes are in bold.

The matrix of geographic distance and the pairwise *F*_ST_ values quantifying genetic differentiation among sample localities are shown, respectively, in Tables S1 and S2. Pairwise estimates of *F*_ST_ varied from 0.446 (between Col – Ata) to 0.837 (between Mar – AgB), and all were statistically significant (*p* < .05), indicating high levels of differentiation between population pairs. The Mantel test showed a high and positive correlation between genetic [*F*_ST_/(1–*F*_ST_)] and geographic distance (*r* = .841, *p* < .002), indicating IBD.

## 4 DISCUSSION

Our study presents no evidence that *P*. *aguadocensis*, *P*. *oliveirana*, *P*. *regentii*, and *P*. *vellozicola* are different species. From the AMOVA analysis, the small proportion of total variance that can be attributed to variation among species strengthens our conclusion that *Pseudolaelia* should be recognized as a single and widely distributed species. In addition, the strong relationship between genetic and geographic distance matrices indicates that historical gene flow has occurred infrequently across the populations.

Although the maximum genetic diversity (i.e., *H*_E_) observable using dominant markers such as ISSRs is 0.5, low levels of within‐population genetic diversity were observed for the PV complex. These values are lower than those reported by Nybom (2004) for plant species with different ecological and life‐history traits. However, high genetic diversity has been reported for orchid species using dominant markers (ISSR and RAPD; Cruz, Selbach‐Schnadelbach, Lambert, Ribeiro, & Borba, 2011; Fajardo et al., 2014; Pinheiro et al., 2012; Ueno, Rodrigues, Alves‐Pereira, Pansarin, & Veasey, 2015). For instance, Fajardo et al. (2014), studying *Cattleya bicolor Lindl*, reported *H*_E_ and *H*_O_ values of 0.219 and 0.323, respectively. Beyond the inheritance of genetic markers, landscape factors due to natural or anthropogenic forest fragmentation, such as the insular distribution of species and geographic isolation of populations, may affect the levels of genetic diversity.

Low levels of genetic diversity similar to those found here for PV complex have also been observed in species that occupy heavily impacted and highly fragmented environments (George, Sharma, & Yadon, 2009; Rodrigues et al., 2015). George et al. (2009) argued that founder effect and genetic bottlenecks resulting from habitat fragmentation as well as limited gene flow due to pollinator behavior are plausible explanations for the low levels of genetic diversity found for *Piperia yadonii*. Rodrigues et al. (2015) suggested that reduced levels of gene flow due to geographical distribution may result in the patterns of low within‐population genetic diversity found for *Cattleya coccinea* and *Cattleya mantiqueirae*. The low level of genetic diversity found herein for the *Pseudolaelia* complex is consistent with the geographical distribution of the species (i.e., populations that are naturally fragmented in the landscape), indicating limited gene flow among populations. In fact, the Mar population, which is the most isolated and located at the highest altitude, showed the lowest values of *H*_E_. Low levels of gene flow and prolonged isolation seems to be a common pattern for populations located in inselberg landscapes worldwide (Barbará, Martinelli, Fay, Mayo, & Lexer, 2007; Boisselier‐Dubayle, Leblois, Samadi, Lambourdière, & Sarthou, 2010; Byrne & Hopper, 2008; Hmeljevski, Nazareno, Bueno, Reis, & Forzza, 2017; Millar, Coates, & Byrne, 2013; Pinheiro et al., 2011, 2014; Tapper et al., 2014a, 2014b). Inselbergs are naturally isolated and fragmented environments not only due to the surrounding vegetation, but also as a result of urban growth and the expansion of farmland (Porembski, 2007), which further undermine the survival of pollinators of species living in outcrops (Barbará et al., 2007). This geographical context could explain the high genetic differentiation found among the studied populations. Furthermore, aspects of reproductive biology (e.g., morphological flower traits and pollination system) are important factors that influence the observed genetic structure.

Although no information about the reproductive biology is available for the PV complex, morphological similarities with *Pseudolaelia geraensis* Pabst, studied by Borba and Braga (2003), can help explain the high interpopulation genetic variation observed herein. *Pseudolaelia geraensis* presents a pollinator deception mechanism that is not a mimetic pair, but rather mimics the general model of melitophilic flowers with nectar guides; however, visits from the bee *Bombus atratus* Franklin are rare and there is a relatively low rate of fruiting. Although the flowers are self‐compatible, the viability of seeds formed by autogamy/geitonogamy is significantly lower than those formed by xenogamy or open pollination. This low viability is the result of inbreeding depression in the early stages of development, leading to embryo abortion. Moreover, the floral morphology of the studied taxa is similar to that observed for *P. geraensis*, with nectar guides and the presence of a narrow‐opening cavity. Thus, there is a possibility that the pollination mechanism of both species is similar. If so, reduced gene flow between populations can be explained by the limited number of pollinator visits, leading to low levels of cross‐pollination and, consequently, high levels of inbreeding. Even with efficient seed dispersal, there is a high probability that seeds are the result of inbred fertilization, with a low germination rate due to inbreeding depression. Moreover, long‐range dispersion is not hampered merely by distance, but also by the need for new individuals to establish in new environments where they must compete for resources and space with other established plants.

Beyond the role of insular geographical distribution and pollination biology of *Pseudolaelia* species, other factors such as mating system, growth form, plant density, and life span can also contribute to lower levels of intrapopulation genetic diversity and the strong genetic structure observed herein. Although no study to date has investigated how these factors affect the genetic diversity of *Pseudolaelia* species, genetic approaches both for orchids and other species with insular distribution have attributed low genetic diversity levels and high *F*_ST_ values to self‐compatibility, limited gene flow, prolonged isolation, and reduced population size (Blambert, Mallet, Humeau, & Pailler, 2016; Hmeljevski et al., 2017; Tapper et al., 2014a, 2014b). These conditions, which are characteristic of inselberg environments, support random genetic drift and limit gene flow and have been suggested as key factors in promoting plant diversification and evolution on terrestrial islands (Hmeljevski et al., 2017; Millar et al., 2013; Pinheiro et al., 2011, 2014; Tapper et al., 2014a, 2014b). Additionally, small effective population sizes seem to be characteristic of epiphytes, which adds to the reduced levels of gene flow and promotes population differentiation and speciation events (Phillips, Dixon, & Peakall, 2012).

Regarding the definition of the species, our results do not support the species delimitation inferred by Castro Neto and Marçal (2007) and Castro Neto and Chiron (2009). In these previous publications, *Pseudolaelia regentii* (Castro Neto & Marçal, 2007) and *P*. *oliveirana* (Castro Neto & Chiron, 2009) were considered distinct species from *P*. *vellozicola*. However, this delimitation was based primarily on highly polymorphic plant and floral morphological characteristics as observed in the field and through linear and geometric morphometric analyses of the floral parts (Menini Neto et al., 2019). In line with recent morphological findings, which are redefining the species boundaries within *Pseudolaelia* (Menini Neto et al., 2019), our population genetics results support the hypothesis that *P. vellozicola* is a highly polymorphic and widely distributed orchid species. As a matter of fact, when we tested the null hypothesis of the existence of only one taxon in the PV complex, we obtained low values of genetic variation among *Pseudolaelia* species (*F*_CT_ = 0.168) indicating that the species of the PV complex are not genetically different. Population genetic analyses based on the repartition of genetic diversity, such as that performed herein, have been used as a good indicator to delimit plant species (Barbará et al., 2007; Bozchaloyi et al., 2018; Després, Gielly, Redoutet, & Taberlet, 2003; Duminil, Hardy, & Petit, 2009; Rodrigues et al., 2015). For instance, Bozchaloyi et al. (2018) used ISSR molecular markers to solve plant species boundaries in the genus *Geranium* (Geraniaceae). Results of that study revealed high values of genetic differentiation among *Geranium* species (*F*_CT _= 0.65) and have served to redefine species limits within the genus (Bozchaloyi et al., 2018).

To conclude, based on the genetic results presented here, and on the morphological findings reported previously by Menini Neto et al. (2019), we propose that all species of the PV complex should be synonymized with *P. vellozicola*. Our study reiterates the importance of population genetics approaches to help solve taxonomic issues in plant species complexes.

## CONFLICT OF INTERESTS

None declared.

## AUTHOR CONTRIBUTIONS

R.C.F. planned the research. L.M.N. and C.V.D.B. collected the samples and identified botanically all trees studied. L.M.N. performed laboratory work. R.S.O.B. and A.G.N. carried out the data analysis. A.G.N., R.S.O.B., L.M.N., and R.C.F. interpreted the data, wrote the manuscript and reviewed the final version.

## STATEMENT OF HUMAN AND ANIMAL RIGHTS

This article does not contain any studies with human participants or vertebrate animals performed by any of the authors.

### Open Research Badges

This article has earned an Open Data Badge for making publicly available the digitally‐shareable data necessary to reproduce the reported results. The data is available at https://doi.org/10.5061/dryad.4mw6m9070.

## Supporting information

AppendixS1

## Data Availability

Gel images of the five ISSR primers showing the scored bands for the sampled individuals and the ISSR raw data for the PV complex are available for download from the Dryad Digital Repository (https://doi.org/10.5061/dryad.4mw6m9070).
