# Supplementary material for: Four raised to one equals one: A genetic approach to the Pseudolaelia vellozicola complex does not follow a math rule
Source: Ecol Evol. 2020 May 5;10(11):4562–9. doi: 10.1002/ece3.6148 (PMC7297771; doi:10.1002/ece3.6148)
Supplement: Supplementary file 1 — AppendixS1 [file ECE3-10-4562-s001.docx]

**Supplementary Material**

**FOUR RAISED TO ONE EQUALS ONE: A GENETIC APPROACH TO THE *Pseudolaelia* *vellozicola* COMPLEX DOES NOT FOLLOW A MATH RULE**

1. ALISON GONÇALVES NAZARENO^1*^, LUIZ MENINI NETO^2^, RENATA SANTIAGO DE OLIVEIRA BUZATTI^1^, CÁSSIO VAN DEN BERG^3^, and RAFAELA CAMPOSTRINI FORZZA^1*^
2. ^1^Department of Genetics, Ecology and Evolution, Federal University of Minas Gerais, Belo Horizonte, Minas Gerais, Brazil.
3. ^2^ Rio de Janeiro Botanical Garden, Rio de Janeiro, Rio de Janeiro, Brazil.
4. ^3^ Department of Biological Sciences, Universidade Estadual de Feira de Santana, Feira de Santana, Bahia, Brazil.

# **Supplementary Figures and Tables**

a

b

**
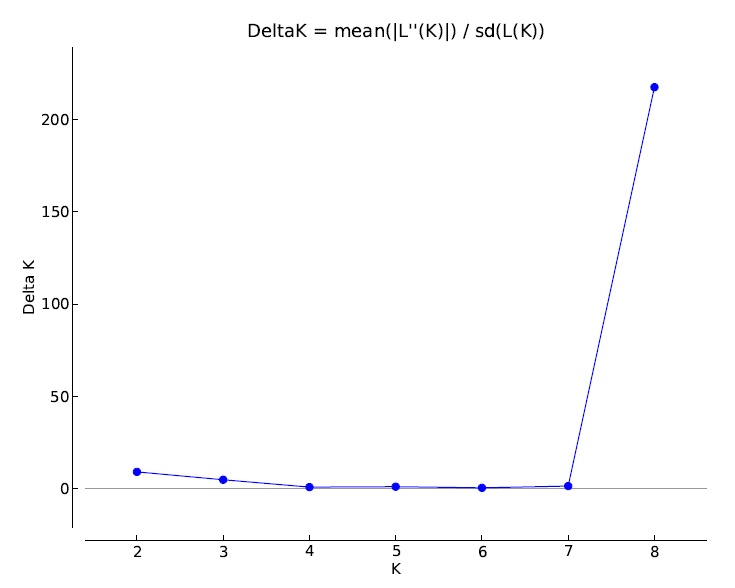
**

**
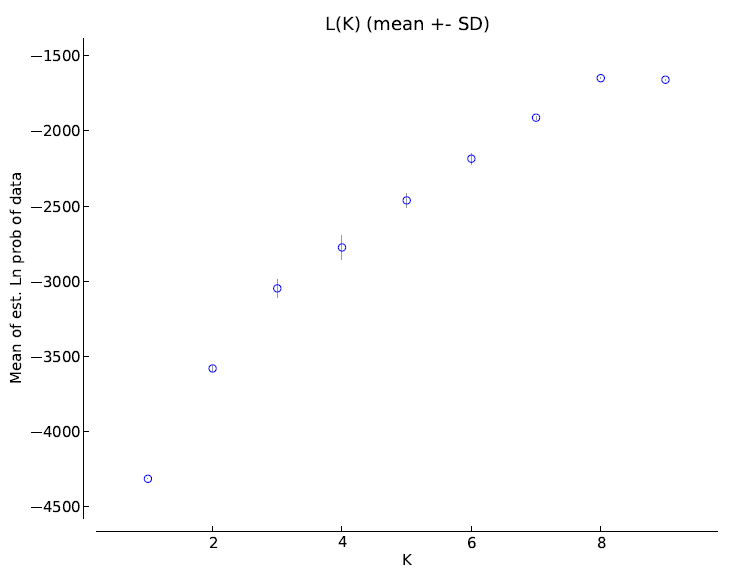
**

**Figure S1.** (a) Log of probabilities LnP(K) and their standard deviation of posterior probability through all runs as suggested by Pritchard *et al.* (2000) and (b) Delta K value estimated using Evanno et al. (2005).

**Table S1.** Matrix of geographical distance (in meters) among all sampled populations.

|  | CamA | CamB | Ata | Col | AgB | AgD | SLe | Mar |
| --- | --- | --- | --- | --- | --- | --- | --- | --- |
| CamA | 0 |  |  |  |  |  |  |  |
| CamB | 1729.222143 | 0 |  |  |  |  |  |  |
| Ata | 419570.4406 | 420930.892 | 0 |  |  |  |  |  |
| Col | 287767.9056 | 289360.1677 | 159276.5395 | 0 |  |  |  |  |
| AgB | 323092.4422 | 324626.6838 | 117283.4342 | 42397.93699 | 0 |  |  |  |
| AgD | 360990.7524 | 362410.6089 | 62589.69781 | 97927.11702 | 57069.69454 | 0 |  |  |
| SLe | 202076.1853 | 203732.8012 | 241967.1669 | 89509.33205 | 129865.706 | 179443.7372 | 0 |  |
| Mar | 933924.0904 | 935322.9867 | 514683.7856 | 656428.3456 | 616090.6957 | 572969.3228 | 745719.4265 | 0 |

**Table S2.** Matrix of genetic divergence (*F*_ST_) among sampled populations

|  | CamA | CamB | Ata | Col | AgB | AgD | SLe | Mar |
| --- | --- | --- | --- | --- | --- | --- | --- | --- |
| CamA | 0 |  |  |  |  |  |  |  |
| CamB | 0.69805 | 0 |  |  |  |  |  |  |
| Ata | 0.63279 | 0.609 | 0 |  |  |  |  |  |
| Col | 0.58047 | 0.60619 | 0.44565 | 0 |  |  |  |  |
| AgB | 0.71675 | 0.74991 | 0.57619 | 0.48082 | 0 |  |  |  |
| AgD | 0.64097 | 0.71228 | 0.5345 | 0.54159 | 0.62817 | 0 |  |  |
| SLe | 0.67562 | 0.71121 | 0.5281 | 0.53836 | 0.60912 | 0.62475 | 0 |  |
| Mar | 0.82817 | 0.82694 | 0.73718 | 0.7367 | 0.83688 | 0.81401 | 0.80907 | 0 |

**References**

Evanno, G., Regnaut, S. & Goudet, J. (2005). Detecting the number of clusters of individuals using the software STRUCTURE: a simulation study. *Molecular Ecology*. 14(8), 2611–2620. DOI: 10.1111/j.1365-294X.2005.02553.x

Pritchard, J. K., Stephens, M. & Donnelly, P. (2000). Inference of population structure using multilocus genotype data. *Genetics*.155(2), 945–959. DOI: 10.1111/j.1471-8286.2007.01758.x
